# CAR-T Cell Therapy: From the Shop to Cancer Therapy

**DOI:** 10.3390/ijms242115688

**Published:** 2023-10-28

**Authors:** Ashanti Concepción Uscanga-Palomeque, Ana Karina Chávez-Escamilla, Cynthia Aracely Alvizo-Báez, Santiago Saavedra-Alonso, Luis Daniel Terrazas-Armendáriz, Reyes S. Tamez-Guerra, Cristina Rodríguez-Padilla, Juan Manuel Alcocer-González

**Affiliations:** Laboratorio de Inmunología y Virología, Facultad de Ciencias Biológicas, Universidad Autónoma de Nuevo León, San Nicolás de los Garza 66450, Nuevo León, Mexico; ana.chavezes@uanl.edu.mx (A.K.C.-E.); cynthia.alvizobz@uanl.edu.mx (C.A.A.-B.); ssaavedraln@uanl.edu.mx (S.S.-A.); luis.terrazasarmn@uanl.edu.mx (L.D.T.-A.); rtamez1804@yahoo.com.mx (R.S.T.-G.); crrodrig07@gmail.com (C.R.-P.)

**Keywords:** CAR-T, TRUCK, cancer therapy, immunotherapy, in vivo CAR-T

## Abstract

Cancer is a worldwide health problem. Nevertheless, new technologies in the immunotherapy field have emerged. Chimeric antigen receptor (CAR) technology is a novel biological form to treat cancer; CAR-T cell genetic engineering has positively revolutionized cancer immunotherapy. In this paper, we review the latest developments in CAR-T in cancer treatment. We present the structure of the different generations and variants of CAR-T cells including TRUCK (T cells redirected for universal cytokine killing. We explain the approaches of the CAR-T cells manufactured ex vivo and in vivo. Moreover, we describe the limitations and areas of opportunity for this immunotherapy and the current challenges of treating hematological and solid cancer using CAR-T technology as well as its constraints and engineering approaches. We summarize other immune cells that have been using CAR technology, such as natural killer (NK), macrophages (M), and dendritic cells (DC). We conclude that CAR-T cells have the potential to treat not only cancer but other chronic diseases.

## 1. Introduction

Cancer remains a significant global health challenge, and one of the leading causes of death worldwide, accounting for 10 million deaths each year [1]. In 2023, 1,958,310 new cancer cases and 609,820 cancer deaths are projected to occur in the United States [2]. However, there have been significant advancements in cancer research and treatment. Early detection, improved diagnostic tools, and better therapies have contributed to increased survival rates for many cancer types. Immunotherapy, targeted therapies, and personalized medicine have emerged as promising approaches to cancer treatment, providing more precise and effective options for patients. Chimeric antigen receptor T-cell therapy (CAR-T cell therapy) is an innovative form of immunotherapy that harnesses the power of the body’s immune system to treat hematological cancer. CAR-T cell therapy has shown remarkable success in treating acute lymphoblastic leukemia (ALL) [3], diffuse large B-cell lymphoma (DLBCL) [4], mantle-cell lymphoma [5], and chronic lymphocytic leukemia (CLL) [6]. 

However, CAR-T cell therapy is not without challenges. Some patients may experience severe side effects such as cytokine release syndrome (CRS) and neurologic toxicities. Additionally, research is ongoing to extend the application of CAR-T cell therapy to solid cancers and to further improve its safety and efficacy. In this context, TRUCKs (T cells redirected for universal cytokine killing) have been developed from work like CAR to produce interleukins that help modulate tumor microenvironments, inducing inflammation and “calling” other T cells into solid tumors. In this paper, we review the latest developments of CAR-T and TRUCKs in cancer treatment. We review part of the history and basics of CAR-T, but also the structure of the different generations and variants of CARs technology. We explain the genetic engineering used to create CAR-T cells ex vivo and in vivo. Moreover, we expose the limitations and areas of opportunity for this immunotherapy and the current trials to treat hematological and solid cancers. Finally, we discuss other immune cells used as CARs. 

## 2. The Bases of Chimeric Antigen Receptor T-Cell (CAR-T) Therapy

Chimeric antigen receptor T-cell therapy is a form of adoptive T-cell immunotherapy (ACT). ACT involves using a patient’s own immune cells, particularly T cells, and enhancing their ability to recognize and attack cancer cells. These engineered T cells are then infused back into the patient to target and eliminate cancer cells more effectively [7]. Currently, there are three forms of ACT: (a) tumor-infiltrating lymphocytes (TILs), which involve tumor-specific T cells from a patient, their expansion in the laboratory, and re-infusing back into the patient to enhance the anti-tumor immune response [8]; (b) the expression of a T-cell receptor (TCR) genetically engineered to recognize a specific cancer antigen [9]; and (c) chimeric antigen receptor T-cell, which involves genetically engineering a patient’s T cells to express a fully synthetic chimeric antigen receptor (CAR) on their surface, allowing more effective recognition and attack of cancer cells [10]. 

CARs are typical, but not exclusive, to T cells. These artificial genetically engineered receptors, called chimeric because are composed of different antibody parts, can be added to an immune effector cell, and enhance its function [11]. CAR employs the binding domain of a monoclonal antibody in a single-chain variable fragment (scFv) format, fused to intracellular T-cell activation domains such as CD28, 4-1BB, OX40, and CD3ζ, replacing the function of the T-cell receptor (TCR), enabling it to directly recognize surface antigens without relying on molecules from the major histocompatibility complex (MHC) for antigen presentation [12]. Indeed, CAR-T is one of the TCR function-independent T cell-based therapeutic strategies employed today [13]. To achieve the creation and clinical use of CAR-T, more than 50 years of research have been undertaken, and strategies continue to be designed and analyzed to improve this therapy against cancer. The main key milestones of CAR-T cells are summarized below.

## 3. The Key Milestones of CAR-T Cells

**Figure 1:**
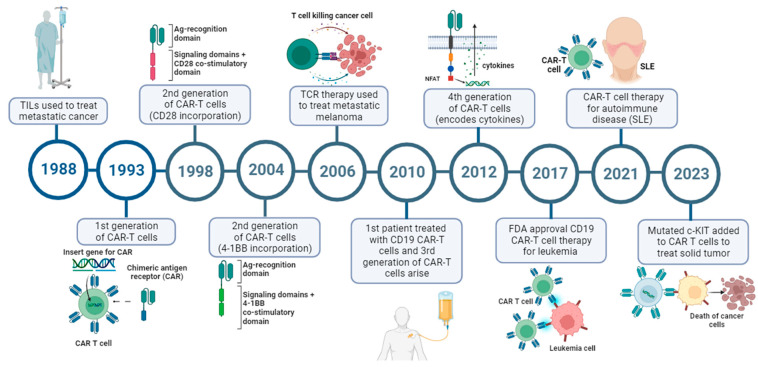
Timeline of CAR-T cell therapy history. The key milestones from the first TIL treatment of metastatic cancer to the newest CAR-T cell therapy application. (This figure was created using BioReder.com).

In the 1960s it was discovered that cells from the immune system could kill cancer cells; from that moment the study of immune cells to treat cancer took off, and the study of T cells as a weapon to fight cancer started. The key milestones in CAR-T history (Figure 1) are explained below:

1. T engineering and ACT: The foundation for CAR-T cell therapy was laid when scientists began exploring ways to engineer T cells to recognize and attack cancer cells. Researchers identified T-cell receptors (TCRs) responsible for recognizing specific antigens present on the surface of target cells. In 1988, the first case of adaptative T immunotherapy using autologous tumor-infiltrating lymphocytes (TILs) was performed by Rosenberg et al. to treat melanoma patients [8].
2. Development of chimeric antigen receptors (CARs): The first CAR design was published in 1993 by Zelig Eshhar [14]. CAR is a recombinant receptor that reprograms T-cell function to target a specific antigen in cancer cells. The in vitro results were promising; however, clinical trials in patients showed low effectiveness for this CAR generation. In 1998, Helen M. Finney described the second CAR-T generation where costimulatory domains (CD28-based) were added into T cells through the CAR construct. This construction enhanced proliferation, cytokine secretion, survival, and anti-tumor activity in T cells [15]. Later, in 2004, another costimulatory domain was proposed (4-1BB), containing constructs that were slower to expand but enhanced persistence in vivo through memory T cells [16]. The third generation emerged in 2010 and comprised two or more costimulatory domains, and the fourth generation arose in 2012. It combined a second-generation CARs backbone with a nuclear factor of activated T cells (NFAT)-driven inducible cytokines [17].
3. First CAR-T Clinical Trials: A pioneer of CAR-T therapies is Dr Carl June from the University of Pennsylvania. In 1997, June’s team used CAR-T in HIV patients; however, it was not until 2010 that he and his colleagues started a trial for humans with leukemia [18] Rosenberg and Kochenderfer’s group treated a patient with chemo-refractory follicular lymphoma (FL) using CD19-CD28 CAR-T cells, resulting in a dramatic regression of tumor infiltration [19]. This encouraging result led to a phase I trial, where eight out of 13 patients reached complete remission (CR). Despite the good results, toxicity was present with cytokine release syndrome (CRS) as well as neurotoxicity [20]. The challenges and setbacks of CAR-T cell therapy are addressed later. After this clinical trial some other trials started, with encouraging results [18,21,22].
4. FDA Approvals (2017–2023): The field of CAR-T cell therapy experienced a breakthrough in 2017 when the U.S. Food and Drug Administration (FDA) approved the first two CAR-T cell therapies: Kymriah and Yescarta. Kymriah (tisagenlecleucel) was developed by Novartis, and targets CD19 for the treatment of relapsed or refractory (r/r) B-cell precursor ALL and r/r large B-cell lymphoma in adults. Yescarta (axicabtagene ciloleucel), by Gilead Sciences/Kite Pharma, also targets CD19 for the treatment of r/r large B-cell lymphoma and r/r follicular lymphoma in adults.

In recent years, four more CAR-T cell therapies have been approved by the FDA.: Tecartus (Brexucabtagene, autoleucel) was approved in 2020 for r/r MCL and r/r B-cell precursor ALL; Breyanzi (Lisocabtagene, maraleucel) was approved in 2021 to treat r/r large B-cell lymphoma, all targeting CD19; Abecma (Idecabtagene vicleucel) was also approved in 2021, targeting BCMA for the treatment of r/r MM; and Carvykti (Ciltacabtagene, autoleucel) was approved in 2022, also targeting BCMA and for the same disease as above [23]. It is important to mention that other regulatory agencies around the world are approving CAR-T cell therapy such as the European Medicines Agency (EMA), the Argentine National Administration of Drugs, Food and Medical Technology (ANMAT), and the Therapeutic Goods Administration (TGA) in Australia.

5. Expanding Applications and Research: After the initial approvals, this technology has been expanded to investigate other blood cancers, solid tumors, and other diseases, such as systemic lupus erythematosus (SLE) [24].

CAR-T therapy is a groundbreaking form of immunotherapy that has shown remarkable success in treating different types of cancer, especially hematological malignancies [25]. However, this therapy presents limited results in solid tumors, including colorectal cancer [26], neuroblastoma [27], and glioblastoma [28]. Moreover, regulatory T cells can infiltrate the tumor, repressing the anti-tumor response and leading to CAR-T cell exhaustion [29]. A better understanding of immune system modulation and tumor microenvironments have led to an improvement in traditional CAR-T cells, resulting in different generations of these CAR-T cells, as explained below.

## 4. The Structure and Generation of CAR-T Cells 

Structurally, CARs are composed of four parts and have undergone stepwise development: 1. the extracellular antigen recognition domain, 2. the hinge or spacer region, 3. the transmembrane domain, and 4. the intracellular component that comprises the T-cell activation domain of CD3ζ, and the signaling domain of costimulatory receptors, which varies depending on the generation of CAR-T. Therefore, the structure dictates the CAR-T generation. 

**Figure 2:**
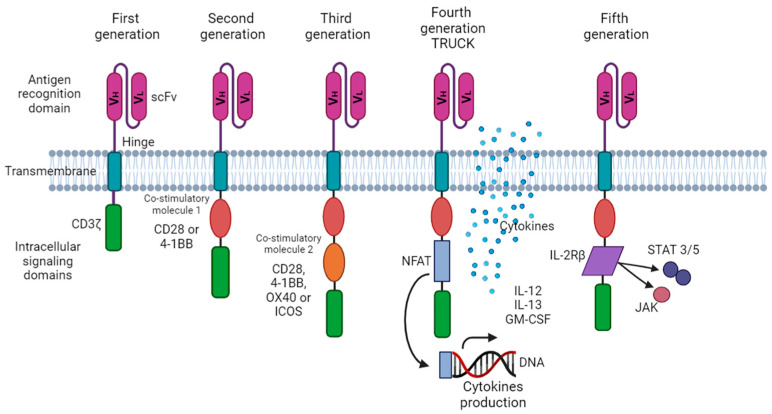
Structure and generation of CAR-T cells. From the first to the fifth CAR-T generation. scFv with the VH and VL forms the antigen recognition domain. The transmembrane domain is depicted as the green rectangle inserted into the lipidic membrane, and the intracellular signaling domains change with each CAR-T generation. Doble chain represent DNA, blue dots cytokines production. (This figure was created at BioRender.com).

Currently, there are five generations of CAR-T cells (Figure 2).

- First CAR-T cell generation: This was the simplest construction, consisting of the extracellular antigen-recognizing domain and the intracellular CD3z, accounting for signal transduction that simulated the natural cellular response [30]. Sadly, the efficiency of this first generation was disappointing because it showed limited persistence of T cells and no anti-tumor effects [31].
- Second CAR-T cell generation: The FDA-approved CAR-T therapies are based on this generation, which differs from the first by having an additional intracellular motif: the signaling domain of costimulatory receptors such as 4-1BB/CD137 or CD28. This extra motif enhances the CAR-T cell persistence in the patient and yields better treatment results [32,33].
- Third CAR-T cell generation: A second costimulatory signaling domain was added to this generation. This modification enhanced T-cell activation, proliferation, and anti-tumor activity [34].
- Fourth CAR-T cell generation: T cells redirected for universal cytokine killing, also known as TRUCKs, are considered “living factories” because they can produce and deliver substances with anti-tumor activity in a specific tissue. These substances are cytokines and other enzymes and costimulatory ligands that enhance T-cell activation [35]. TRUCKs will be discussed further in a separate section below.
- Fifth CAR-T cell generation: These are based on the second generation of CARs, but they contain a truncated cytoplasmic IL-2 receptor β-chain domain with a binding site for the transcription factor STAT3. The antigen-specific activation of this receptor simultaneously triggers TCR (through the CD3ζ domains), co-stimulatory (CD28 domain), and cytokine (JAK–STAT3/5) signaling, which effectively provide all three synergistic signals (TCR engagement, co-stimulatory signaling, and cytokine signaling) that are physiologically required to drive full T-cell activation and proliferation [36].

**Figure 3:**
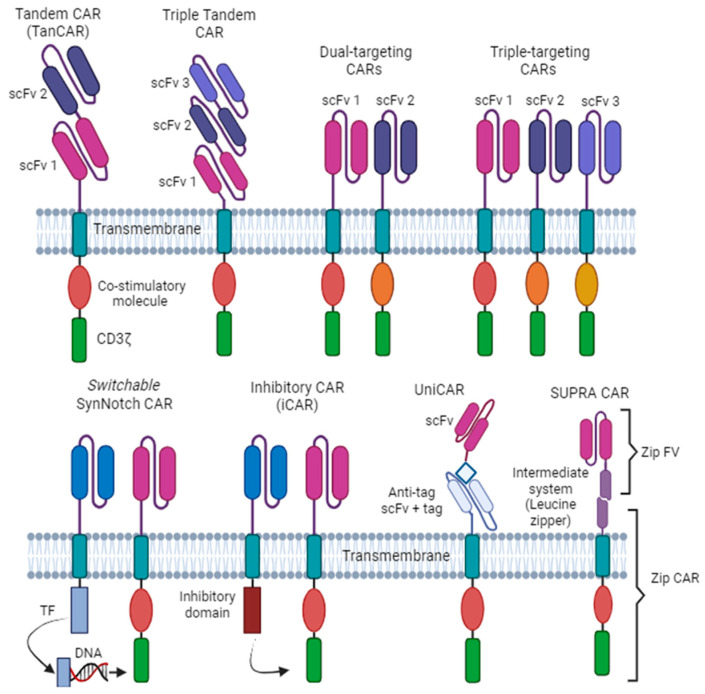
Variants of CARs and generations of CAR-T cells. Tandem CAR (TanCAR), Triple Tandem CAR (3 scFv), double CAR, triple CAR, CAR synNotch, inhibitory CAR (iCAR), universal CAR (uniCAR), and split universal programmable (SUPRA) CAR. scFv, single-chain variable fragment; TF, transcriptional factor.

Additionally, variants have been designed to enhance the specificity, accuracy, killing capability, antigen recognition, and safety of CAR-T cells even in controlled tumor microenvironments (Figure 3), such as:

- The Tandem CAR (TanCar), which uses two scFvs for two different tumor surface antigens connected to a single transgenic receptor [37]. Each generation of CAR-T can be adapted to TanCAR.
- Dual-targeting CARs, which can also recognize two different antigens simultaneously, but they are engineered in two separate CARs on the same T cell, with each CAR cell having its own intracellular domains [38].
- The Triple scFvs in tamdem [39], which is like TanCar but uses three scFvs. However, its construction and improvements are very complicated. Also, it can be three separate CARs as in dual-CARs.
- Switchable, AND-gate, inhibitory CARs, which incorporate a safety mechanism that allows for the control of CAR-T cell activity. The switch can be activated to turn off CAR-T cells in case of severe side effects or unwanted toxicities. Gate CARs use the SynNotch receptor (modular receptor), and CAR-T cells are only activated when two scFvs bind to corresponding tumor antigens simultaneously (the synNotch and the scFvs) [40]. Inhibitory CARs (iCARs) contain a traditional CAR and an iCAR on the surface of a T cell [41]. The iCAR recognizes non-tumor cells and enables them to inhibit the T-cell killing signaling. Then, CAR-T cells function normally, but when iCARs bind to the target non-tumor antigens, inhibition of traditional CARs occurs, protecting non-tumor cells from damage.
- Universal CARs and Universal CAR-T Cells. Universal CAR-T cells pretend to increase the range of antigen recognition, using a “third party” intermediate system between the transmembrane domain and the scFv (examples include Universal CARs, BBIR CAR, and SUPRA CAR). Universal CAR-T cells are designed to be derived from healthy donors, and are modified to avoid immune rejection when infused into different patients. These “off-the-shelf” CAR-T cells have the potential to provide readily available and cost-effective treatments. For more information on special CARs and Universal CARs see Miao et al. (2022) and Zhao et al. (2018) [42,43].

Because TRUCKs are among the newest generations of CAR-T cells, we dedicate the next section to explaining this CAR-T generation. 

## 5. T Cells Redirected for Universal Cytokine Killing (TRUCKs)

As mentioned, TRUCKs are the fourth generation of CAR-T cells. These cells were developed in 2011 and represent a significant advancement in the field of CAR-T cell therapy since incorporating transgenic cytokine production and release. TRUCKs with proinflammatory interleukins like IL-7, IL-12, IL-18, IL-21, and IL-23 are under active research because the innate immune cells are attracted and activated by these cytokines, helping tumor destruction by exocytosis (perforin, granzyme) or death ligand–death receptor (Fas–FasL, TRAIL) systems [36]. TRUCKs were developed to induce a proinflammatory milieu in solid tumors, helping to modulate the tumor microenvironment with the release of cytokines [29]. TRUCKs, which release IL-12 [44], IL-15 [45], IL-18 [46], IL-21 [47], and IL-23 [48], were successfully used within in vitro and in vivo models, whereas other cytokines are currently being explored as well. The cytokine is delivered locally in the tumor stroma, inducing acute inflammation and leading to a comprehensive anti-tumor response in tumor-bearing mice [49]. 

The way TRUCKs work is by using CAR-T technology modified with an inducible expression system for the protein of interest, allowing them to specifically target tumor tissue and deliver a desired transgenic protein, particularly a cytokine upon CAR engagement of the cognate antigen, and signaling to the targeted area. This system is controlled by 6xNFAT-responsive elements and the IL-2 minimal promoter. When the CAR is activated through CD3-ZAP70 signaling and boosted by co-stimulation, it triggers NFAT activation, leading to the expression of the transgenic protein via the NFAT/IL-2 minimal promoter. This system can provide stimulation either in an autocrine manner, supporting their own survival and proliferation, or in a paracrine fashion, modulating the immune cell environment [29]. Proinflammatory cytokines applied systemically induce severe toxicities. This problem is solved with TRUCKs, because the interleukin is only released upon CAR engagement with the antigen. When CAR-T cells are no longer in contact with the cognate antigen, the interleukin production stops as a switch-off, making it safe. This CAR-T generation has been tested in glioblastoma, neuroblastoma, and pancreatic and lung tumors in pre-clinical experiments [36,50]. Moreover, novel and automatized manufacturing of TRUCKs targeting specific overexpressed molecules like disialoganglioside (GD2) in cancer cells has been developed, making it possible to treat different tumors using the same technology [35].

Even though this technology is very promising for the treatment of cancer, there are limitations (which will be discussed next), as well as engineering that is being investigated to overcome these limits in its design.

## 6. Genetic Engineering to Create CAR-T Cells Ex Vivo and In Vivo

**Figure 4:**
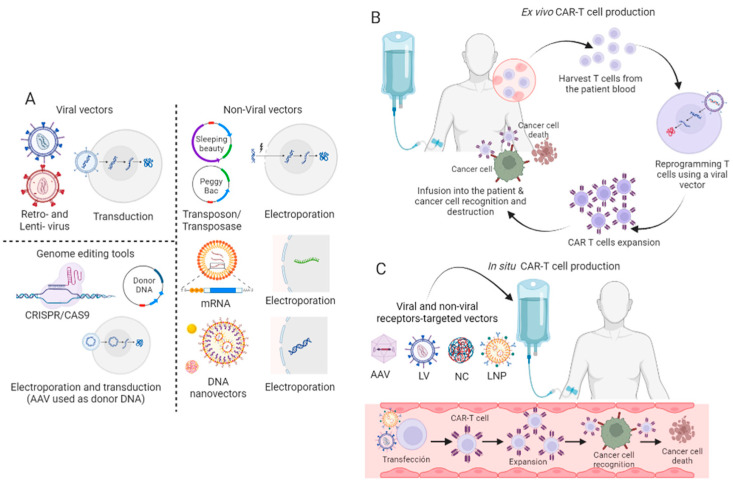
Genetic engineering to create CAR-T cells ex vivo and in situ. (**A**) Vectors used to genetically modify T cells to express CAR. Ex vivo and in vivo (in situ) CAR-T cell manufacturing. (**B**) Ex vivo CAR-T production involves isolating a patient’s T cells, ex vivo reprogramming, expansion, infusion into the patient’s bloodstream, and tumor cell destruction. (**C**) In vivo CAR-T production involves using nanocarriers loaded with CAR genes and gene-editing tools. The nanocarrier systems include polymer nanoparticles and viral vectors which reach and edit T cells in situ at tumor sites to kill cancer cells. (This figure was created using BioRender.com).

Currently, CAR-T cells can be manufactured ex vivo and in vivo. The main challenges of CAR-T production are the high cost and scale manufacturing limitation [51]. Ex vivo CAR-T production involves isolating a patient’s T cells, ex vivo reprogramming to target cancer cells, expansion, infusion into the patient’s bloodstream, and tumor cell destruction. To build a CAR-T cell, different approaches have been used (Figure 4), such as viral and non-viral vectors including transposon/transposase systems and mRNA electroporation, nonintegrating DNA nano-vectors, and genome editing tools like CRISPR Cas-9. In vivo, CAR-T production refers to the strategy of engineering CAR-T cells directly within the patient’s body. In this approach, the genetic material required to express the CAR is delivered directly into the patient’s T cells in vivo, allowing them to be transformed into CAR-T cells within the body itself. The in vivo method seeks to simplify the manufacturing process and avoid graft–versus–host disease (GvHD) [51]. Next, we offer a brief explanation of CAR-T cell tool production ex vivo and in vivo.

Viral vectors, especially retroviruses, and lentiviruses, are used for CAR-T cell engineering due to their high transduction efficiency. Nevertheless, limited genetic cargo, high costs of production, and the safety concern regarding the use of viruses that can help tumor development due to insertional mutagenesis are obstacles to general clinical translation [52]. However, all CAR-T cell therapies approved by the FDA to date use retroviruses or lentiviruses for genetic engineering. The development of viral vectors for clinical use in CAR-T cell technology is reviewed in Irving et al. (2021) [53]. 

Transposons are units of DNA that can change their position within the genome. The DNA transposon has two functional components: the terminal inverted repeats (TIRs) and the transposase. Sleeping Beauty (SB) DNA transposon is capable of transposition in human cells and is safer than Piggy Back (PB); for those reasons it has been used in some early clinical trials exploring CAR T cell therapy [54]. Transposons are more cost-effective, less toxic, and can facilitate the co-delivery of multiple genes compared to viral systems. Although the SB platform has promising applications in cancer immunotherapy, its use has been limited by the low efficiency of plasmid DNA delivery into primary human cells [55]. 

mRNA electroporation has been used for T-cell modification. Electroporation uses pulsed high-voltage electrical currents to transiently create small pores in the cell membrane, allowing nanometer-sized cargo to enter the cell. Some advantages of mRNA electroporation are fast and cost-effective manufacturing, and the fact that the patient can be infused 24 h post-electroporation, in comparison with the 10- to 14-day manufacturing time needed for virus- or transposon-engineered CAR-T cells. mRNA electroporation has been used in clinical trials and has been shown to be safe, but with a non-important clinical response [53].

CRISPR Cas-9 is a two-component system, composed of a single-stranded guide RNA (sgRNA) and a Cas9 endonuclease. There is a preoccupation that CRISPR/Cas9 gene editing could promote tumor malignancy due to off-target mutagenesis, or cause immunogenicity from an anti-Cas9 response. However, this technology offers the potential for simultaneous multiple loci editing [55]. Recently, more studies using this system have been applied successfully in patients with relapsed/refractory B-cell non-Hodgkin lymphoma (r/r B-NHL) [52].

In vivo, CAR-T cells use nanocarriers loaded with CAR genes and gene-editing tools. The nanocarrier systems include polymer nanoparticles and viral vectors that are then targeted to tumor regions in vivo to edit T cells in situ at tumor sites to kill neoplastic cells. Smith et al. (2017) showed encouraging results for regressing leukemia using synthetic DNA nanocarriers [56]. Later, the same group designed an RNA nanocarrier for transiently reprogramming circulating T cells to recognize different antigens. They demonstrated that repeated infusions of these polymer nanocarriers induce the regression of lymphocytes in human leukemia and prostate cancer, and hepatitis B-induced hepatocellular carcinoma in a mouse model [57]. In addition to the polymer nanoparticles, viral vectors such as lentiviruses and adenovirus-associated viruses (AAV) have also been tested for the in vivo generation of CAR-T cells [58,59,60]. Moreover, the use of CAR-T in vivo is not exclusive to treating cancer. Cardiac fibrosis was treated with anti-fibrotic CAR-T cells in vivo by delivering modified mRNA in T cell-targeted lipid nanoparticles. Treatment with modified mRNA-targeted lipid nanoparticles reduced fibrosis and restored cardiac function in a mouse model of heart failure [61]. In vivo, CAR-T generation can enhance the targeted killing of tumor cells and reduce systemic toxicity, and protocol can be standardized easily [62]. Additionally, in vivo, genetic engineering of CAR-T cells can be used to treat other health problems. 

In vivo CAR-T production is active research, with no FDA-approved product yet. However, the pre-clinical results show less complication than ex vivo production. We address the main complications of CAR-T cell therapy next. 

## 7. Complications of CAR-T Therapy

As mentioned before, CAR-T therapy can target various cell-surface molecules without the need for antigen processing or HLA presentation, making it suitable for patients with different HLAs [10,63]. Nevertheless, advances in the use of this therapy for the treatment of malignancies have been reported, and its application has been associated with severe adverse reactions, some of which are life-threatening for the patients. The most reported complications during CAR-T cell therapy administration include cytokine release syndrome, neurotoxic symptoms, off-target damage, anaphylaxis, secondary infections, tumor lysis syndrome, B-cell dysplasia, coagulation disorders, and cytopenia [64].

Cytokine release syndrome (CRS), also known as “cytokine storm”, is the most common adverse reaction to CAR-T cell therapy. Its incidence in patients with hematological malignancies receiving CAR-T therapy is approximately 55.3%, with severe CRS affecting about 18.5% of cases [65,66]. Mild CRS symptoms include fever, fatigue, headache, joint pain, and myalgia, while severe cases can lead to hypotension, high fever, shock, vascular leakage, DIC, and MODS [67]. Treatment plans for CRS vary based on its grade, ranging from symptomatic treatment for grade 1 to supportive care and immunosuppression for higher grades [68].

The second most frequent adverse effect associated with CAR-T therapy is immune effector cell-associated neurotoxicity syndrome (ICANS). It is characterized by neurotoxic symptoms caused by the activation of T cells and other immune cells. The reported incidence of ICANS in patients with hematological malignancies receiving CAR-T therapy ranges from approximately 21.7% to 37.2% [64]. Symptoms include reduced attention, language and writing disorders, confusion, lethargy, tremors, and in severe cases, seizures, motor weakness, elevated intracranial pressure, and cerebral edema [69]. ICANS pathogenesis may involve elevated levels of certain cytokines and increased permeability of the blood–brain barrier [68,70]. Early prevention and intervention of CRS may help reduce the occurrence of ICANS. Therapeutic regimens for ICANS include fasting, nutritional support, improved neurological examination, and administration of tocilizumab or siltuximab [71]. 

Off-target effects refer to the negative effects of CAR-T cell therapy resulting from the killing of normal cells expressing targeted antigens along with tumor cells. Increasing the specificity of CAR-T cells by optimizing their structure can help reduce off-target effects, but finding new tumor-specific antigens (TSAs) is challenging and costly [64]. Furthermore, anaphylaxis has been observed during CAR-T cell therapy, likely caused by the presence of murine monoclonal antibody-derived antigen recognition domains in CAR-T cells. This can lead to IgE-mediated anaphylactic events [40,41,72]. 

Infections associated with CAR-T cell therapy are relatively common in patients, and factors contributing to infection risk include severe CRS and/or CRES, long-term use of glucocorticoids, CAR-T cell-induced B-cell dysplasia and hypogammaglobulinemia, high-grade CRS, and treatment with stronger anti-tumor drugs [68,73].

Tumor lysis syndrome (TLS) can occur during CAR-T cell therapy due to the rapid necrosis of tumor cells, leading to the release of intracellular substances and metabolites into the blood. It is more common in hematological malignancies, particularly large volume and high metabolism tumors [74]. 

Some hematological pathologies have been observed during CAR-T cell therapy administration. For example, targeting CD19 can lead to B-cell dysplasia, as CD19 is highly expressed not only on malignant B cells but also on benign and most normal B cells [75,76]. Hemophagocytic lymphohistiocytosis (HLH) and macrophage activation syndrome (MAS) are clinical syndromes associated with excessive inflammation and cytokine release, with HLH characterized by abnormal proliferation of lymphocytes and tissue cells and MAS by excessive activation of T cells and macrophages [77,78,79]. Also, coagulation disorders are of common occurrence during CAR-T cell therapy, with various coagulation-related abnormalities observed, such as increased D-dimer, fibrinogen degradation products, prolonged prothrombin time, decreased fibrinogen, and thrombocytopenia [80]. 

Finally, cytopenia, which includes neutropenia, thrombocytopenia, and anemia, is a common adverse reaction to CAR-T cell therapy. The incidence varies by disease types and treatment approaches, and supportive care and anti-infective treatments are recommended for management [5,81]. 

To enhance the safety of CAR-T cell therapy, efforts should focus on minimizing the impact of these adverse reactions, allowing more patients to benefit from this treatment.

## 8. Clinical Linkage of CAR-T Cells

**Figure 5:**
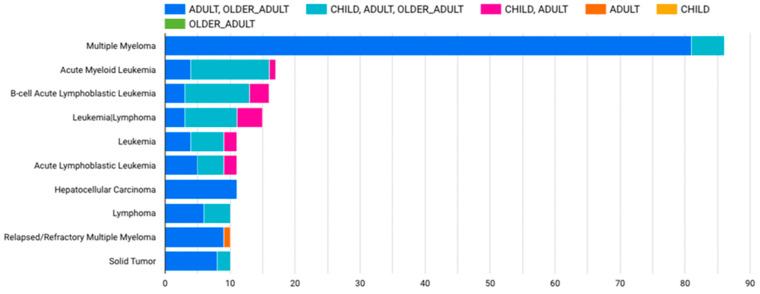
Current clinical trials according to the age of participants. We performed a search on clinicaltrials.gov using the keyword “cancer” as the condition and “CAR-T cell therapy” as the intervention/therapy, filtering by “recruiting” as the study status. A list of 448 different studies was retrieved. The raw data were downloaded as a CSV file and then analyzed using Google data analysis tools to build the graphs. Most of the clinical trials for CAR-T cell therapy registered on clinicaltrials.gov include adult and older adult patients.

In 2012, the field of cellular immunotherapy was relatively young when the first pediatric patient received CTL019, now known as Tisacel, for CAR-T therapy. Since then, there has been a significant increase in trial activity, with nearly 100 new trial registrations every year (Figure 5) [82]. Currently, the ClinicalTrials.gov website has more than 1000 registered clinical trials for CAR-T cell therapy in cancer patients, using “CAR-T” as a keyword search. The top 15 of these clinical trials are summarized in Table 1.

Clinical trials have explored CAR-T cells targeting various tumor-associated antigens. Below, we describe several studies focusing on these target-specific CAR-T cells.

Tisacel contains the 4-1BB co-stimulatory domain, whereas other CAR-T products like KTE-X19 use the CD28 co-stimulatory domain. The 4-1BB co-stimulatory domain CAR-T has demonstrated more durable in vivo persistence compared to the CD28 co-stimulatory domain CAR-T. [83] Trials have differed in CAR vector constructs, manufacturing, eligibility criteria, patient population, and dosing schemes. However, despite these variations, CD19 CAR-T therapy has consistently shown high complete remission rates in high-risk, heavily pretreated patients with relapsed or refractory B-cell acute lymphoblastic leukemia (R/R B-ALL).

CAR-T cells specific to Her-2, also known as human epidermal growth factor receptor 2 (Her-2, neu, or ErbB2), belong to the epidermal growth factor receptor (EGFR) family. Her-2 is a well-researched tumor-associated antigen in cancer immunotherapy, given its widespread expression on various cancer cells, including medulloblastoma, osteosarcoma, glioblastoma, and breast cancer cells. Initial studies involving N29—a first-generation CAR based on HER2-specific scFv—demonstrated its specificity and ability to lyse cancer cells dependent on the presence of HER2. Another CAR known as FRP5 led to sustained regression of established medulloblastomas in a mouse model [28]. Subsequent investigations confirmed the functionality of second-generation (CD28) CARs, expressing canine T cells and targeting Her-2 in osteosarcoma [84]. Further research revealed that modified CD28 s-generation CAR-T cells effectively hindered tumor growth in mice with osteosarcoma or breast tumor xenografts. Overall, these findings underscore the promising potential of Her-2 targeted CAR-T therapy for clinical applications [85]. 

The oncogenic variant of the epidermal growth factor receptor, known as EGFRvIII, is not present in normal tissue but is exclusively expressed in tumors of several cancer types, including glioblastoma (GBM), breast cancer, and non-small cell lung cancer (NSCLC). Most studies focusing on redirecting CAR-T cells to target EGFRvIII were conducted using glioblastoma models. Early preclinical investigations demonstrated the potential anti-tumor effects of first-generation CARs designed to target EGFRvIII [86,87]. Subsequent studies involving second- and third-generation CAR-T cells further validated their anti-tumor activity against EGFRvIII-positive glioblastoma cells. Moreover, when third-generation CAR-T cells were directly injected into the brain, they successfully inhibited tumor growth [88]. Interestingly, after systemic administration, third-generation CAR-T cells targeting EGFRvIII were able to localize to intracranial tumors, suppress tumor growth, and improve the survival of mice with established GBM xenografts [89].

Mesothelin is a tumor-associated antigen found highly expressed in various cancer types, including malignant pleural mesotheliomas (MPM), pancreatic cancers, and ovarian cancers. However, it is only minimally expressed on normal peritoneal, pleural, and pericardial mesothelial surfaces [90]. As a result of this differential expression, immunotherapy targeting mesothelin has shown promising outcomes. For instance, one study involved engineering T cells with a strong affinity for mesothelin (CD28-41BBz) and administering them either intratumorally or intravenously into immunodeficient mice. These mice had tumors established using primary pleural effusion cells from mesothelioma patients. The results showed that the engineered T cells effectively reduced the tumor burden and, in some cases, completely eradicated the tumors at low effector-to-target ratios [91]. 

Similarly, another study in 2010 demonstrated that intratumoral injection of RNA CAR electroporated T cells led to the regression of large, vascularized flank mesothelioma tumors in mice [92]. It is important to note that these T cells were found to be less potent in this model compared to lentivirus-transduced T cells, which effectively cured most mice. Additionally, Lanitis conducted an adoptive transfer of mesothelin-targeted first-generation CAR-T cells in a xenogeneic model, which resulted in a regression of ovarian cancer [93]. These findings collectively support the promising clinical potential of mesothelin-specific CAR-T cells for the treatment of malignant pleural mesotheliomas, ovarian cancer, and other solid tumors.

GD2-specific CAR-T cells are designed to target ganglioside GD2, which is a surface antigen commonly found in various human cancers and stem cells, including pediatric embryonal tumors and adult cancers. Due to its limited presence in normal tissues, GD2 is considered a safe target for immunotherapy [94]. The third-generation CAR-T cells engineered with the GD2-specific antibody sc14.G2a showed significant cytokine secretion upon recognizing the antigen, and demonstrated effective anti-melanoma activity in both laboratory tests and xenograft models [95]. Moreover, first-generation CAR-T cells co-expressing the chemokine receptor CCR2b were developed to enhance their homing capabilities, leading to improved anti-tumor activity against GD2+ neuroblastoma-secreting CCL2 [96].

CEA, a 180-kD tumor-associated glycoprotein, is well-known for its role as a tumor-inducer and is found to be overexpressed in various epithelial cancers, particularly in colorectal adenocarcinoma and pancreatic tumors. A recent study demonstrated the rejection of CEA-positive pancreatic tumors in CEA-transgenic mice, where the mice expressed CEA as a self-antigen in healthy gastrointestinal cells [97]. The use of first-generation CAR-T cells targeting CEA proved effective in eliminating CEA+ tumors during the initial response. Furthermore, cured mice displayed a robust and sustained recall response against CEA+ tumor cells upon rechallenge. Additionally, the anti-tumor response of CD28-costimulatory CAR-T cells was validated in an animal model of CEA+ colorectal cancer [98].

NK cells are well-known for using activating receptors like NKG2D, NKp30, and DNAX accessory molecule-1 (DNAM-1) to identify stress-induced ligands present in different tumor types. As a result, CARs containing the ligand-binding region of these receptors or the single-chain variable fragment (scFv) against these ligands were developed, and these CAR-T cells were assessed in preclinical animal models, showing encouraging anti-tumor outcomes [99,100]. However, there were instances of lethal toxicity observed [100]. Apart from these commonly targeted receptors, numerous other antigens are emerging as potential CAR-T targets for solid tumors. Other studies have indicated that interleukin-13Ra2 specific CAR-T cells could potentially be used to eliminate glioblastoma [101]. Overall, existing preclinical data strongly support the notion that CAR-T cells exhibit powerful anti-tumor properties.

In addition to these commonly used targets, other antigens are emerging as potential CAR-T targets in solid tumors. Preclinical data strongly support the robust anti-tumor activity of CAR-T cells.

Future trials are needed to address challenges in identifying response predictors, improving the risk–benefit balance, and reducing the financial burden for patients and healthcare systems. Applying trial response rates to real-world patients is complex, as inclusion criteria often favor patients with better prognoses. Comparative analysis of patient cohorts with varying disease burdens has revealed that patients with high disease burdens tend to have inferior outcomes compared to those with lower or no detectable disease before CAR-T infusion.

## 9. Non-CAR-Ts: CAR-Natural Killers, CAR-Macrophages, and CAR-Dendritic Cells

Thanks to our increasing knowledge of the immune system, CAR genetic engineering, and the possibility to improve new cells to obtain better results than CAR-T cells for cancer and other immune diseases, cells like natural killers (NK), macrophages, and dendritic cells (DC) are in active research. 

NK cells have innate anti-tumor properties. In human peripheral blood, NK cells represent around 5% to 15% of the leukocytes. NK cells are functionally similar to cytotoxic T cells. However, NK killing is independent of HLA, and an important antibody-dependent cell-mediated cytotoxicity (ADCC) mediator [102]. CAR-NKs were created by Tran in 1995, offering an alternative platform for cancer immunotherapy. Some advantages that CAR-NK presents are that they are easier to obtain than NK cells, they do not require HLA compatibility, and they have limited toxicity [103]. Moreover, these immunotherapies not only kill cancer cells in a CAR-dependent manner, but also independently. Currently over 70 clinical trials are underway using CAR-NK cells against cancer [104] (Supplementary Table S1). The NK92 cell line, human peripheral blood mononuclear cells (PBMCs), umbilical cord blood, and induced pluripotent stem cells (iPSCs) can all be used to produce “off-the-shelf” CAR-NK products [101]. Each of the NK sources has advantages and disadvantages. For more information about manufacturing and clinical trials, consult [102,103].

Macrophages are phagocytic immune cells that can either promote tumor growth and progression or participate in anti-tumor immune responses. By modifying macrophages with CARs, researchers aim to skew their behavior toward an anti-tumor phenotype, improving their ability to recognize and eliminate cancer cells. CAR-M was introduced for the first time by Klichinsky in 2020 [105]. The advantages of CAR-M are good presence in solid tumors, phagocytosis, and tumor-antigen presentation [106,107]. Currently, four clinical trials are underway using CAR-M cells against cancer. (Supplementary Table S2). Like in CAR-NK, the sources of macrophages are PBMCs, iPSCs, and cell lines like THP-1. Also, a CAR-M targeting HER2-positive solid tumors has been developed with encouraging results. However, the main challenge of CAR-M therapy is the fact of macrophages cannot proliferate by themselves. For more information, see [108]. 

Dendritic cells are a type of antigen-presenting cell (APC) that plays a vital role in initiating and regulating the immune response by presenting antigens to T cells [108]. developed CAR-DC for the first time. CAR-DCs can cross-present antigens and prime T cells to respond against new antigens. The cDC1 subset is particularly effective at initiating de novo T-cell responses, potentially overcoming the problem of antigen escape seen in other CAR therapies [109]. Currently, three clinical trials are underway using CAR-DC cells against cancer. (Supplementary Table S3).

## 10. CAR-T Cell Therapy Constraints & Engineering Approaches

CAR-T cells represent a novel category of treatment and mark a fresh therapeutic approach. The adaptable structure of existing CARs allows for enhancements tailored to tackle the unique challenges presented by the tumor microenvironment (TME) in specific diseases. Through the use of synthetic biology and gene-editing tools, scientists can adeptly modify CAR-T cells to enhance safety and efficacy. 

Various innovative engineering techniques have been employed to enhance the effectiveness of CAR-T cells. CAR-T cell products engineered to target multiple tumor-associated antigens (TAAs) can counteract antigen escape or heterogeneity. Modifications based on this approach involve creating CAR-T cells that simultaneously produce and secrete bi-specific T cell engagers (BiTEs) or developing CARs that focus on adapter molecules, which can be linked to diverse soluble antigen-recognition components, enabling the recognition of multiple antigens with just one CAR. Augmenting the in vivo persistence of CAR-T cells can be achieved by using less differentiated T cell subsets or by integrating factors into CAR-T cells that foster a supportive microenvironment, such as 4-1BB ligand (4-1BBL) [110].

The migration and penetration of CAR-T cells within solid tumors can be enhanced by equipping these cells to respond to chemokines associated with tumors or by targeting physical barriers within the tumor microenvironment (TME). Lastly, CAR-T cells can be engineered to surmount the immunosuppressive elements found in the TME [111]. This can involve bypassing the effects of inhibitory immune checkpoints, like programmed cell death 1 (PD-1) or inducing an inflammatory environment through the expression of cytokines or other immune-stimulating factors like CD40 ligand (CD40L).

Significantly, the increased complexity of CAR designs and the genetic modification of T cells may enhance the potential risks linked to CAR-T cell therapy. For instance, the use of viral transduction and gene-editing tools brings about the possibility of unintended gene disruption outside the intended target, a phenomenon known as off-target effects. The transformation of T cells into malignant clones, resulting from insertional mutagenesis that can activate proto-oncogenes or disrupt tumor-suppressor genes, stands as a well-anticipated theoretical hazard of gene therapy. While no instances of such transformation through insertional mutagenesis have been reported in patients so far, clinical observations have noted viral insertions into genes in patients treated with lentiviral transduced CAR-T cells for chronic lymphocytic leukemia (CLL) [112]. Specifically, the CAR gene was integrated into the TET2 loci, leading to the clonal expansion of T cells [112]. Although this clonal T cell population eventually diminished naturally, this occurrence underscores the risk associated with treating patients using genetically altered cells. Nonetheless, as researchers accumulate more experience with adoptive cell transfer, their confidence in managing this risk is growing. This is reflected in the initiation of a pioneering human trial aimed at assessing a transgenic TCR T cell product directed against NY-ESO-1, which has undergone multiple CRISPR–Cas9 gene edits to eliminate the native TCR and PD-1.

The intricate genetic modification of cellular products outside the body also introduces complexity into the manufacturing process. Experience gained from the current procedure of CAR-T cell manufacturing has revealed that during the collection of lymphocytes and circulating tumor cells in the apheresis product, inadvertent transduction of the CAR construct can occur. This unintentional transduction can lead to a situation where CARs present on these tumor cells bind to tumor-associated antigens (TAA) on the same cells, causing antigen masking [113]. This, in turn, results in the clonal expansion of these cells and their escape within the body. It is noteworthy that CAR-T cell technology stands as the pioneer among clinically approved gene therapies. Ongoing surveillance of complications associated with gene editing in trials involving CAR-T cell products will contribute to a deeper understanding of the long-term risks associated with the emerging field of gene editing in medical applications [113]. This monitoring effort could potentially aid in devising strategies to address and mitigate these complications.

## 11. Conclusions and Perspectives

CAR-T cell therapy has great potential to combat advanced leukemias and lymphomas; researchers are very optimistic about this type of therapy. However, this immunotherapy is limited in solid tumors because activated myeloid cells secrete inflammatory cytokines, such as IL-6 and IL-1β, that lead to the inflammatory toxicities observed in patients infused with CAR-T cells [51,52,53]. In addition, the modulation occurring in the tumor microenvironment such as immunosuppressive cytokines, regulatory T-cell infiltration and lack of CAR-T infiltration is a challenge for the researchers to improve CAR-T effects [51,114]. Many investigations have demonstrated that when CAR-T cell therapy is employed in conjunction with various cancer treatments like radiotherapy, chemotherapy, anti-cancer vaccines, oncolytic viruses, BiTEs, cytokines, checkpoint inhibitors, immunomodulators, and metabolic inhibitors, it results in a synergistic impact of these combined therapies, enhancing the therapy outcomes since both can reduce tumors and provide a microenvironment that helps CAR-T cell infiltration [115]. Indeed, some drugs can break immunosuppression, allowing cytotoxic immune cells to attack tumors. Chemotherapeutic drugs like cyclophosphamide and fludarabine are used prior to CAR-T cell therapy but only to deplete lymphocytes to avoid cell engraftment. Nowadays, chemotherapy is being used as adjuvant treatment with CAR-T, in preclinical and clinical trials of the drugs doxorubicin, docetaxel, oxaliplatin, and several more [115,116]. However, challenges are still occurring since chemotherapeutic drugs are toxic and have many side effects. The use of PD-1 blockades in combination with CAR-T in different studies shows some encouraging outcomes of combinations. CAR-T cells are also being studied to treat chronic diseases such as Alzheimer’s [117], Parkinson’s [118], and lupus [24].

The application of this therapy includes several stages of development such as manufacture, manipulation, and delivery to the patient. This is a great challenge for the health system, so it must be sustainable with fully qualified personnel to maximize efficiency in routine clinical practice and to provide safety for the patients who acquire it, as we mention before in “Genetic Engineering to Create CAR-T Cells Ex Vivo and In Vivo”. Undoubtedly CAR-T cell therapy has a promising future, and although currently all treatments are autologous, there is a great interest in developing this therapy to be universal, with “on-the-shelf” availability of CAR-T to treat not only cancer but other chronic diseases. 

## Figures and Tables

**Table 1:** Current top 15 recruiting studies on CAR-T cell therapy.

	NCT Number	Study Title	Conditions
**1**	NCT05963217	Study of TBI-2001(Autologous CD19 Specific Chimeric Antigen Receptor (CAR) Gene-transduced T Lymphocytes) for Relapsed or Refractory CD19+ B-cell Lymphoma, CLL/SLL	Relapsed or Refractory CD19+ B-cell Lymphoma|Relapsed or Refractory Chronic Lymphocytic Leukemia|Relapsed or Refractory Small Lymphocytic Lymphoma
**2**	NCT05963100	Clinical Study of TCR-like CAR-T Cell Targeted MSLN in the Treatment of Ovarian Cancer	Safety and Efficacy of TCR-like CAR-T
**3**	NCT05952375	Exploratory Clinical Trial on the Safety, Efficacy, and Pharmacokinetics of XKDCT086 (iPD-1-Claudin18.2-CAR-T) in Claudin 18.2 Positive Advanced Solid Malignant Tumors: a Single Center, Single Arm, Dose-increasing Trial	Gastric Cancer
**4**	NCT05950802	Optimizing Lymphodepletion to Improve Outcomes In Patients Receiving Cell Therapy With Yescarta	DLBCL—Diffuse Large B Cell Lymphoma
**5**	NCT05948033	CD70 Targeted CAR-T Cells in CD70 Positive Relapsed/Refractory Lymphoma	Lymphoma
**6**	NCT05947487	CD70 Targeted CAR-T Cells in CD70 Positive Advanced/Metastatic Solid Tumors	Solid Tumor, Adult
**7**	NCT05941156	Clinical Study of Anti-CD56-CAR-T in the Treatment of Relapsed/Refractory NK/T Cell Lymphoma /NK Cell Leukemia	Extranodal NK T Cell Lymphoma|NK-Cell Leukemia
**8**	NCT05932173	A Study of Novel Anti-CD19 CAR-T in Patients With r/r B-Cell Malignancies	B-Cell Leukemia|B-Cell Lymphoma|B-cell Tumors
**9**	NCT05926726	GPC3-directed CAR-T in the Treatment Amongst Subjects With Advanced Hepatocellular Carcinoma	Hepatocellular Carcinoma
**10**	NCT05923541	RD13-02 for Patients With r/r CD7+ T Cell Hematologic Malignancies	Hematologic Malignancies
**11**	NCT05902845	RD13-02 CAR-T Cell Injection for Patients With r/r CD7+ T-ALL/T-LBL	Neoplasms|Hematologic Neoplasms|Hematologic Diseases
**12**	NCT05891197	A Biomarker Screening Protocol for Participants With Solid Tumors	Triple Negative Breast Cancer|Nonsmall Cell Lung Cancer
**13**	NCT05878184	Study Evaluating SC291 in Subjects With r/r B-cell Malignancies (ARDENT)	Non Hodgkin Lymphoma|Chronic Lymphocytic Leukemia
**14**	NCT05875402	XKDCT080 in GCC-positive Recurrent or Refractory Solid Tumors With Single Center, Single Arm, and Dose Escalation	Colon Cancer
**15**	NCT05873712	Zanubrutinib and Lisocabtagene Maraleucel for the Treatment of Richter’s Syndrome	Recurrent Transformed CLL, Refractory Transformed CLL, Transformed CLL to Diffuse Large B-Cell, Lymphoma/Recurrent Transformed B-Cell Non-Hodgkin, Lymphoma/Recurrent Transformed Non-Hodgkin

We performed a search on clinicaltrials.gov using the keyword “cancer” as the condition and “CAR-T cell therapy” as the intervention/therapy. Filtering by “recruiting” as the study status, a list of 448 different studies was retrieved.

## Data Availability

Not applicable.

## Supplementary Materials

The supporting information can be downloaded at: https://www.mdpi.com/article/10.3390/ijms242115688/s1.
