# Supplementary material for: CAR-T Cell Therapy: From the Shop to Cancer Therapy
Source: Int J Mol Sci. 2023 Oct 28;24(21):15688. doi: 10.3390/ijms242115688 (PMC10649325; doi:10.3390/ijms242115688)
Supplement: Supplementary file 1 [file ijms-24-15688-s001.zip › ijms-2576194-supplementary.pdf]

Supplementary

	NCT Number ▾	Study Title	Conditions
1.	NCT06006403	Safety and Efficacy of CD123-targeted CAR-NK for Relapsed/Refractory Acute Myeloid Leukemia or Blastic Plasmacytoid Dendritic Cell Neoplasm	Acute Myeloid Leukemia|Blastic Plasmacytoid Dendritic Cell Neoplasm (BPDCN)|Relapse Leukemia|Refractory Leukemia
2.	NCT05995028	Universal 4SCAR7U Targeting CD7-positive Malignancies	T-cell Acute Lymphoblastic Leukemia|T-cell Acute Lymphoblastic Lymphoma|Acute Myeloid Leukemia|NK Cell Lymphoma
3.	NCT05987696	Natural Killer(NK) Cell Therapy in Acute Myeloid Leukemia	AML, Adult|Minimal Residual Disease
4.	NCT05941156	Clinical Study of Anti-CD56-CAR-T in the Treatment of Relapsed/Refractory NK/T Cell Lymphoma /NK Cell Leukemia	Extranodal NK T Cell Lymphoma|NK-Cell Leukemia
5.	NCT05922930	Study of TROP2 CAR Engineered IL15-transduced Cord Blood-derived NK Cells Delivered Intraperitoneally for the Management of Platinum Resistant Ovarian Cancer, Mesonephric-like Adenocarcinoma, and Pancreatic Cancer	Pancreatic Cancer|Ovarian Cancer|Adenocarcinoma
6.	NCT05856643	Single-arm, Open-label Clinical Study of SZ011 in the Treatment of Ovarian Epithelial Carcinoma	Ovarian Epithelial Carcinoma
7.	NCT05845502	Single-arm, Open-label Clinical Study of SZ003 in the Treatment of Advanced Hepatocellular Carcinoma	Advanced Hepatocellular Carcinoma
8.	NCT05842707	Study of Cord Blood-derived CAR NK Cells Targeting CD19/CD70 in Refractory/Relapsed B-cell Non-Hodgkin Lymphoma	Refractory or Relapsed B-cell Non-Hodgkin Lymphoma
9.	NCT05776355	NKG2D CAR-NK & Ovarian Cancer	Ovarian Cancer
10.	NCT05739227	Safety and Efficacy of Allogenic CD19-CAR-NK Cells in Treatmenting r/r B-cell Hematologic Malignancies	Acute Lymphoblastic Leukemia|B-cell Lymphoma|Chronic Lymphocytic Leukemia
11.	NCT05703854	Study of CAR.70-engineered IL15-transduced Cord Blood-derived NK Cells in Conjunction With Lymphodepleting Chemotherapy for the Management of Advanced Renal Cell Carcinoma, Mesothelioma and Osteosarcoma	Advanced Renal Cell Carcinoma|Advanced Mesothelioma|Advanced Osteosarcoma
12.	NCT05686720	Single-arm, Open-label Clinical Study of SZ011 in the Treatment of Advanced Triple Negative Breast Cancer	Advanced Triple Negative Breast Cancer
13.	NCT05673447	The Study of Anti-CD19 CAR NK Cells in the Treatment of Relapsed/Refractory Diffuse Large B Cell Lymphoma	Diffuse Large B Cell Lymphoma
14.	NCT05667155	Clinical Study of Cord Blood-derived CAR NK Cells Targeting CD19/CD70 in Refractory/Relapsed B-cell Non-Hodgkin Lymphoma	B-cell Non Hodgkin Lymphoma
15.	NCT05654038	A Study of Universal CD19-Targeted UCAR-NK Cells Combined With HSCT for B Cell Hematologic Malignancies	B-Cell Lymphoblastic Leukemia/Lymphoma

Supplementary Table S1. Clinical trials for CAR-NK application in cancer patients. A search was performed at clinicaltrials.gov using “cancer” as a condition query and “CAR-NK cells” for Intervention/treatment. We retrieved 70 studies applying CAR-NK cells therapy alone or in conjunction with other treatments for solid and hematologic cancers, the table shows top 15 registered trials.

	NCT Number	Study Title	Conditions
1.	NCT05007379	Cohort Study to Determine the Antitumor Activity of New CAR-macrophages in Breast Cancer Patients' Derived Organoids	Breast Cancer
2.	NCT04660929	CAR-macrophages for the Treatment of HER2 Overexpressing Solid Tumors	HER2-positive|Adenocarcinoma|Bile Duct Cancer|Biliary Tract Cancer|Bladder Cancer|Breast Cancer|Breast Neoplasm|Carcinoma, Ductal|Carcinoma, Hepatocellular|Cancer|Lung Cancer, Non-Small-Cell|Carcinoma, Ovarian Epithelial|Carcinoma, Small Cell|Carcinoma, Squamous|Carcinoma, Transitional Cell|Colorectal Cancer|Esophagogastric Junction Neoplasms|Inflammatory Breast Cancer|Stomach Neoplasms|Malignant Neoplasms|Ovarian Neoplasms|Pancreatic Cancer|HER2-positive Solid Tumors|HER2-positive Breast Cancer|HER2-positive Gastric Cancer|HER-2 Protein Overexpression|HER-2 Gene Amplification|Prostate Cancer|Head and Neck Cancer|Endometrial Cancer|Lung Cancer, Small Cell
3.	NCT04314843	Study of Lenzilumab and Axicabtagene Ciloleucel in Participants With Relapsed or Refractory Large B-Cell Lymphoma	Relapsed/Refractory Large B-cell Lymphoma
4.	NCT03827343	Retrospective Study of Immunotherapy Related Toxicities and Factors Impacting Outcomes in Children and Adults With Cancer	Macrophage Activation Syndrome|Primary Hemophagocytic Lymphohistiocytosis
5.	NCT03392545	Combination of Immunization and Radiotherapy for Malignant Gliomas (InSituVac1)	High Grade Glioma|Glioblastoma|Glioma of Brainstem|Glioma, Malignant

Supplementary Table S2. Clinical trials for CAR-macrophages application in cancer patients. A search was performed at clinicaltrials.gov using “cancer” as a condition query and “CAR-macrophages” for Intervention/treatment. We retrieved 5 studies applying CAR-macrophages therapy alone or in conjunction with other treatments for solid and hematologic cancers.

	NCT Number	Study Title	Conditions
1.	NCT05631899	Combination of CAR-DC Vaccine and ICIs in Local Advanced/Metastatic Solid Tumors	Solid Tumor, Adult|EphA2 Overexpression|KRAS G12V|KRAS G12C|KRAS G12D
2.	NCT05631886	Combination of CAR-DC Vaccine and ICIs in Malignant Tumors	Solid Tumor, Adult|Lymphoma|EphA2 Overexpression|TP53 R273H|TP53 R175H|TP53 R248Q|TP53 R249S
3.	NCT05585996	Exploratory Clinical Study of CD19-targeted CAR-T and CAR-DC in the Treatment of Relapsed and Refractory B-cell Lymphoma	Relapsed and Refractory B-cell Lymphoma
4.	NCT05277753	NGS-MRD Assessment of Combination Immunotherapies Targeting T-ALL	T-Cell Acute Lymphoblastic Leukemia
5.	NCT05262673	NGS-MRD Assessment of Combination Immunotherapies Targeting B-ALL	B-Cell Acute Lymphoblastic Leukemia

Supplementary Table S3. Clinical trials for CAR-DC (CAR-dendritic cells) application in cancer patients. A search was performed at clinicaltrials.gov using “cancer” as a condition query and “CAR-DC” for Intervention/treatment. We retrieved 5 studies that applied CAR-DC therapy alone or in conjunction with other treatments for solid and hematologic cancers.
